# Microcystic Stromal Tumor with Predominant Bizarre Nuclei of Ovary in a Pregnant Woman

**DOI:** 10.1155/2022/8457901

**Published:** 2022-12-07

**Authors:** Tip Pongsuvareeyakul, Chalita Kingnate, Kornkanok Sukpan, Surapan Khunamornpong

**Affiliations:** ^1^Department of Pathology, Faculty of Medicine, Chiang Mai University, Chiang Mai, Thailand; ^2^Department of Obstetrics and Gynecology, Lamphun Hospital, Lamphun, Thailand

## Abstract

Microcystic stromal tumor (MST) is a rare type of pure stromal tumor in the category of ovarian sex cord-stromal tumors. It is characterized by a distinctive microcystic appearance with bland tumor cells. Although the pathological diagnosis can be straightforward based on the typical histomorphology in most MSTs, the cases with morphologic variation can pose a diagnostic challenge due to unfamiliarity of pathologists with the histologic spectrum of MST and its negativity for inhibin and calretinin, the commonly used sex cord-stromal markers. The coexistence between MST and mucinous epithelial tumor is extremely rare. We present the first case, to our knowledge, of ovarian MST with predominant bizarre nuclei coexisting with mucinous cystadenoma in a pregnant woman. The histomorphology in this case presents a diagnostic challenge and raises differential diagnosis for a wide variety of ovarian malignant neoplasms including nonneoplastic lesions.

## 1. Introduction

Microcystic stromal tumor (MST) is a rare type of pure stromal tumor of the ovary, with less than 50 reported cases in English literature [1]. It was first described by Irving and Young in 2009 [2]. The tumor shows monotonous bland nuclei with distinctive morphology of 3 components namely: microcystic spaces, solid cellular areas, and hyalinized fibrous stroma, with a characteristic immunophenotype including aberrant diffuse nuclear expression of beta-catenin, diffuse positivity for CD10, and negative staining for the widely used sex cord-stromal immunomarkers such as inhibin and calretinin [2, 3]. Bizarre nuclei can be found but usually only with focal [2]. To our knowledge, the combination of MST and mucinous tumor of the ovary has not been previously reported.

Herein, we report the first case of ovarian MST with predominant bizarre nuclei and the coexistence with mucinous cystadenoma in a pregnant woman. Unusual morphologic alteration of MST and the presence of mucinous epithelial component pose a diagnostic challenge to pathologists and may result in misclassification of tumor and overdiagnosis.

## 2. Case Presentation

A 27-year-old pregnant Asian woman with gestational age of 38 weeks by ultrasound was admitted for cesarean section. The patient had a history of gestational diabetes mellitus type A2. An ultrasound scan showed an incidental left ovarian cyst. Her family histories were unremarkable. The patient underwent cesarean section with left salpingo-oophorectomy. The patient remains alive and well without evidence of disease 69 months after surgery.

### 2.1. Pathologic Examination

**Figure 1:**
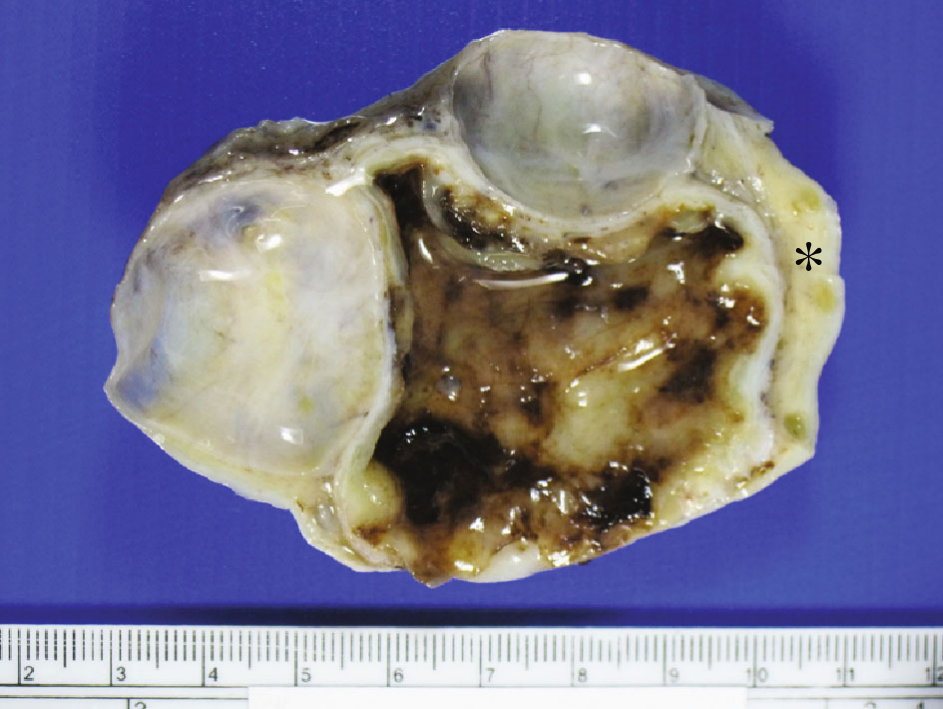
Cut surface of the ovary shows three cystic lesions which are well-demarcated from the remaining ovarian parenchyma (asterisk). The largest locule has nodular white lining with focal hemorrhage, whereas the other two locules show smooth internal surface.

**Figure 2:**
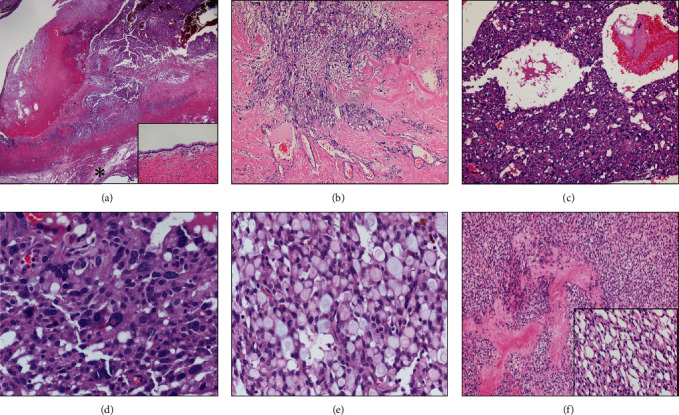
Microcystic stromal tumor (MST) of the ovary. (a) Solid nodule with infiltrative border, separate from a mucinous cyst (asterisk) by thick fibrous stroma (H&E, 1.25×). The cyst is lined with benign mucinous epithelium (inset) (H&E, 20×). (b) The irregular infiltrative-like border of MST (H&E, 10×). (c) Solid pattern with microcysts of variable caliber and the diffuse presence of bizarre cells. Large spaces containing blood and fibrin are also observed (H&E, 10×). (d) Bizarre cells with epithelioid features and pleomorphic hyperchromatic nuclei (H&E, 40×). (e) Aggregates of signet ring-like cells (H&E, 40×). (f) Typical MST features of cellular areas with hyalinized fibrous bands (H&E, 10×). Monotonous tumor cells with bland nuclei punctuated by relatively uniform microcystic spaces (inset) (H&E, 40×).

**Figure 3:**
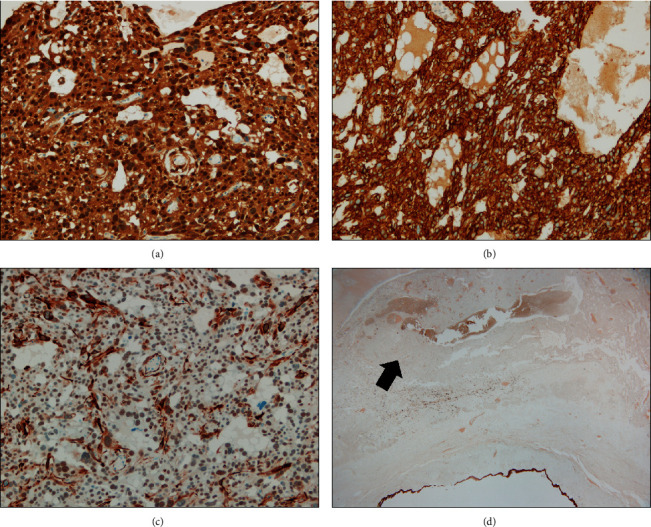
Immunohistochemical stains of MST. (a) Diffuse, strong nuclear and cytoplasmic expression of beta-catenin (20×). (b) Diffuse, strong cytoplasmic positivity for CD10 (20×). (c) Patchy positivity for WT-1 (20×). (d) EMA negativity in MST (arrow). Note the positive staining in mucinous cystadenoma (lower) (1.25×).

The left ovarian mass measured 8.5 × 7 × 5 cm and revealed smooth external surface. The cut surface showed three abutting cystic lesions, which were well-demarcated from the remaining rim of ovarian parenchyma (Figure 1). The largest locule was 4.5 cm with nodular and hemorrhagic internal surface and thick white wall. The other two locules were round with smooth internal surface and measured 2.5 and 3 cm in diameter. Microscopically, we examined a total number of 19 tissue sections. Two histologically distinct tumor types were identified. These components were separated by ovarian stroma without mixed or transitional area (Figure 2(a)). The largest locule was lined by fibrinous material or sheet-like growth of neoplastic cells of varying thickness with focal nodular thickening. This component had an irregular border characterized by an infiltrative-like interface with surrounding fibrous ovarian stroma (Figure 2(b)). Tumor cells were arranged in predominant solid pattern with occasional microcystic spaces of variable caliber (Figure 2(c)). Microcysts focally coalesced to form larger spaces containing blood with fibrinous material. Bizarre cells with enlarged hyperchromatic nuclei or multinucleation were diffusely distributed and were present in approximately 80% of tumor areas (Figure 2(d)). Cells with intracytoplasmic vacuoles (signet ring-like) were also mingled with these bizarre cells (Figure 2(e)). Typical areas of MST were focally observed, characterized by microcystic architecture with hyalinized fibrous stroma (Figure 2(f)) and uniform bland, round-to-oval tumor nuclei with fine chromatin and pale to eosinophilic cytoplasm (Figure 2(f), inset). Mitotic figures were not detected. The two cystic locules with smooth internal surface were lined by benign mucinous epithelium (Figure 2(a), inset). The fallopian tube was unremarkable. Immunohistochemically, tumor cells in the largest locule showed diffusely strong nuclear and cytoplasmic reactivity for beta-catenin (Figure 3(a)), diffuse positivity for CD10, patchy positivity for WT-1 (Figures 3(b) and 3(c)), and focal expression of CK (AE1/AE3), whereas the immunostains for inhibin, calretinin, AFP, CK7, and EMA were negative (Figure 3(d)). The mucinous component was positive for CK, CK7, and EMA (Figure 3(d), arrow) and showed normal membranous staining for beta-catenin. The diagnosis was ovarian microcystic stromal tumor with predominant bizarre nuclei coexisting with mucinous cystadenoma.

## 3. Discussion

The coexistence between MST and epithelial tumor of the ovary is extremely rare. To our knowledge, this is the first report of the combination of ovarian MST and mucinous tumor. Furthermore, the presence of predominant bizarre nuclei has not been reported in MST, and the tumor was diagnosed during pregnancy. This extremely rare occurrence accompanied by the unusual histomorphology may cause diagnostic confusion with ovarian malignant tumors, either primary or metastatic, or even nonneoplastic lesions.

In this case, the tumor showed diffuse cytologic atypia and an irregular infiltrative-like interface with ovarian stroma. It was focally immunopositive for CK and negative for the commonly used sex cord-stromal markers such as inhibin and calretinin. The morphologic and immunophenotypic features may raise a concern for anaplastic carcinoma mural nodule arising in ovarian mucinous tumor. Moreover, the presence of signet ring-like cells may lead to a suspicion of the Krukenberg tumor. It is important to note that mitotic figures were not observed, and the immunostaining for other epithelial markers such as EMA and CK7 was negative. Based on these features, the possibility of epithelial cancer is less likely.

Germ cell tumor should also be included in differential diagnosis as the ovarian tumor occurred in a young adult patient. The morphologic features may be confused with yolk sac tumor that usually contains irregular meshwork of spaces merging with cysts of varying size and exhibits high-grade nuclear atypia. Focal CK positivity without the expression of EMA and CK7 can be found in YST. In this context, a lack of mitosis together with negativity for AFP supports the exclusion of YST. As mentioned earlier, nonneoplastic lesions related to hormonal effects during pregnancy may also be included in the consideration. Luteinized follicular cyst of pregnancy and puerperium typically appears as cystic mass and may contain bizarre cells with few or no mitoses. However, the absence of luteinized granulosa cells in the cystic lining including the negativity for inhibin and calretinin are helpful clues to rule out this diagnosis.

Although MST is typically immunonegative for inhibin and calretinin, the tumor is immunoreactive for SF-1 and FOXL-2 [3] that has been recently identified to be specific markers of sex cord-stromal differentiation [4]. However, these immunomarkers may not be available in many pathology laboratories. In the present case, not only the negativity for inhibin and calretinin but also the focal staining of CK may potentially mislead pathologists away from the recognition of the sex cord-stromal nature. The important clue to the diagnosis of MST in our case is the presence of typical characteristic morphologic features, although focal, confirmed by typical immunoprofile of beta-catenin, CD10, and WT-1. It is noteworthy that CK positivity can be found in approximately 45% of sex cord-stromal tumors, particularly in Sertoli-Leydig cell tumors [5].

Bizarre nuclei can be found in approximately 60% of MST, but these cells account for less than 5-10% of overall tumor volume in most cases [2]. MST with diffuse bizarre cells, as seen in this case, may potentially lead to overlooking of MST features and overestimation of the malignant potential of the tumor. Predominant bizarre nuclei have not been reported in MST; however, in a report by He et al., bizarre nuclei were present in up to 50% of tumor with an uneventful follow-up outcome [6]. Bizarre cells in MST may represent degenerative changes similar to those seen in other sex cord-stromal tumors and their presence does not appear to affect prognosis [2, 7]. Bizarre nuclei can also be seen in some gynecological tract lesions (such as uterine smooth muscle tumors and vaginal polyp) and these changes have been associated with pregnancy or progestin therapy [7]. Whether the diffuse presence of bizarre nuclei in MST in this case is related to hormones in pregnancy remains to be clarified.

The association between MST and mucinous epithelial component may be explained by a coexistence of two unrelated tumors with different histogenetic origins (collision tumor) or by that mucinous component representing a heterologous element in ovarian sex cord-stromal tumors like those seen in granulosa cell tumor and Sertoli-Leydig cell tumor [8, 9]. In this case, the tumor was extensively sampled, and we could not identify a transitional area between MST and mucinous cystadenoma. Moreover, mutation of beta-catenin, which is involved in the pathogenesis of MST, has not been encountered in mucinous cystadenoma [10]. This is supported by our IHC results that the mucinous component showed different expression pattern from MST. We therefore hypothesize that the association of MST and mucinous cystadenoma in this case is most likely coincidental.

Genetic alterations in the Wnt/beta-catenin pathway play a crucial role in tumorigenesis of MST [3, 11]. Mutation of beta-catenin (CTNNB1) gene is found in 73% of MST, whereas the minority of cases exhibits a mutually exclusive mutation in APC gene [11, 12]. Both genes are involved in Wnt/beta-catenin pathway and their mutation can result in aberrant nuclear expression of beta-catenin [12]. Thus, invariably nuclear staining for beta-catenin together with diffuse positivity of CD10 can be used as confirmation immunostaining for MST [11, 12]. Interestingly, 80% of MST containing APC mutation exhibited clinical features of familial adenomatous polyposis (FAP) [11]. It is noteworthy that MST with mutated APC may be an extracolonic manifestation of this syndrome [11, 12]. Although the definitive conclusion has not been reached, in the clinical viewpoint, the recognition of ovarian MST may draw an attention for further clinical screening and genetic testing for FAP [11]. Unfortunately, the testing was not available in this case.

Although the majority of MST patients had a benign clinical course, the understanding of the clinical behavior of this tumor may be limited by its rarity and the lack of follow-up in some cases [2]. Recently, two cases of MST with recurrence or extraovarian spread have been reported [13, 14]. Tumors in both cases were found in the reproductive age group and exhibited classical morphologic and immunophenotypic features. In one patient, recurrence was detected in bilateral ovaries and bilateral iliac fossa 9 years after cystectomy for MST [13]. In the other patient, microscopic spread in the omentum was detected at the diagnosis of MST [14]. These findings may raise the possibility for the uncertain malignant potential of this tumor [13, 14]. Further molecular study of additional cases is required to warrant the biologic and clinical behavior of MST.

In conclusion, we present the unique case of ovarian MST with diffuse bizarre nuclei coexisting with mucinous cystadenoma in a pregnant woman. The extremely rare occurrence may pose a diagnostic challenge to pathologists. Awareness of these unusual features together with a thorough sampling of tumor and the judicious use of immunostains will aid the correct diagnosis.

## Data Availability

The histological and immunohistochemical data used to support the findings of the study are included within the article.
